# How perceptions of bone marrow donation costs affect donation behavior: survey evidence from a large donor registry

**DOI:** 10.1007/s10198-025-01785-4

**Published:** 2025-05-27

**Authors:** Michael Haylock, Patrick Kampkötter, Mario Macis, Susanne Seitz, Robert Slonim, Edith Wienand, Daniel Wiesen, Alexander H. Schmidt

**Affiliations:** 1 CINCH Essen, University of Duisburg-Essen, Essen, Germany; 2 Faculty of Management, Economics and Social Sciences, Eberhard Karls University Tübingen, Nauklerstraße 47, 72074 Tübingen, Germany; 3 Johns Hopkins University, Carey Business School, IZA, and NBER, Baltimore, USA; 4 DKMS Group gGmbH Tübingen, Kressbach 1, 72072 Tübingen, Germany; 5 University of Technology Sydney and IZA, Sydney, Australia; 6 University of Cologne, Department of Health Care Management and Erasmus University Rotterdam, Erasmus School of Health Policy and Management, Rotterdam, Netherlands

**Keywords:** Unrelated stem cell donation, Information, Stem cell extraction methods, Perceived costs, Availability, I12, I18

## Abstract

Over the past three decades, advancements in collection methods for hematopoietic stem cell transplantation substantially reduced invasiveness and safety concerns. To what extent, however, registered donors are informed about extraction methods and how their beliefs drive their willingness to follow through with a donation is not well understood. Inaccurate beliefs about extraction methods may cause donors to overestimate their perceived cost, potentially reducing donations. In a survey with about 24,000 potential donors in Germany’s largest stem-cell registry, we investigate how beliefs about extraction methods affect potential donors’ willingness to follow through with a stem cell donation. We find widespread misconceptions about extraction methods, with many donors attributing a significant fraction of stem cell extractions to be coming from never-used methods. Importantly, a lack of knowledge and misconceptions about extraction methods persist among registered donors, often anchored to methods that prevailed at the time of registration. Exploring the link between donors’ beliefs and their (stated) willingness to donate, we find that accurate beliefs about lower extraction costs correlate with a 2.2–2.9 percentage points higher willingness to donate, representing a 40% reduction in donor unavailability. Our results highlight the need for informational campaigns to correct donors’ misconceptions and potentially save more lives among blood cancer patients.

## Introduction

Hematopoietic stem cell transplantation (HSCT) from an unrelated donor has extended the lifespan of hundreds of thousands of patients worldwide and enhanced their quality of life (e.g., [1, 9]). However, at least one fifth of individuals registered as potential stem cell donors do not follow through with a donation. They are unavailable at the so-called confirmatory typing (CT) stage, a crucial milestone in the process of donating stem cells (e.g., [3, 10]).^1^ At this stage, a potential donor who is found to be a close match for a patient is asked whether they wish to proceed with the donation process or not. High rates of unavailability of potential donors at this stage can (i) prolong the time until a suited donor who is willing to donate is found, or, more severely, (ii) reduce the likelihood that a stem cell collection is actually carried out. As a result, these two incidences may negatively affect the likelihood of patient survival [12]. Understanding the reasons why potential donors do not follow through with a donation is therefore of utmost importance for patients and the registry management alike.

One reason for potential donors’ unwillingness to donate, which has been insufficiently addressed in the literature so far, is donors’ beliefs about the stem-cell extraction methods. Different methods, such as extraction from the bloodstream or from the pelvis, may be perceived to vary in costs due to their differing levels of invasiveness and associated risks, which can influence donors’ propensity to donate. Besides increasing the benefits of making donations, which has been extensively explored (e.g., [11]), the potential of lowering the perceived costs of donating to increase the likelihood of making a donation has not been sufficiently investigated. Craig et al. [2] show, for example, that donors experiencing longer wait times to make blood donations, causing them to believe that they would have to give up more time (i.e., a higher cost) to make their next donation, resulted in fewer donations overall. More generally, standard economic theory predicts that the higher potential donors expect the costs of donating to be, the less likely they will (*ceteris paribus*) be to make the donation. There are many possible costs in the context of making a stem cell donation. Here, we focus on costs regarding the potential pain and risks of stem collection methods, which may affect registry members’ likelihood to follow through with a donation.

In this study, we examine potential donors’ understanding of stem cell extraction methods. We identify two common misconceptions about stem cell donations: (i) stem cells are extracted from the spine, and (ii) a surgical procedure is always needed. However, both beliefs are wrong and suggest that some donors likely believe the donation process is potentially more risky and painful (e.g., surgery versus no surgery) than it actually is. To date, the evidence supporting these misconceptions is primarily anecdotal. Nonetheless, there is reason to believe that these misconceptions could be an important barrier to stem-cell donor follow-through at CT, given newspaper articles and stem-cell donor registries’ measures to counteract these misconceptions by describing them as “myths.”^2^

We make two main contributions. Our first contribution is to comprehensively assess the knowledge about stem cell extraction methods among actual potential donors in a major donor registry. Our second and most novel contribution is to investigate the extent to which misconceptions about these methods correlate with stem-cell registry members’ willingness to donate. We particularly explore whether incorrect beliefs—such as the assumption that donating stem cells is a more painful or risky procedure than it actually is—might have detrimental effects on donors’ propensity to be available when called upon to donate. More specifically, we address the following research questions: (i) How well-informed are prospective donors about the extraction methods currently used in stem cell donation? (ii) Do potential donors’ beliefs about stem cell extraction methods relate to their willingness to donate stem cells?

We answer these questions through a large online survey of about 24,000 prospective stem cell donors living in Germany conducted with DKMS, a major international stem cell donor registry. Currently, there are two possible methods of stem cell extraction: peripheral blood stem cell donation and bone marrow donation. Peripheral blood stem cell collection occurs from the bloodstream via peripheral veins and is similar to a hemodialysis procedure. Prior to collection, stem cells are “mobilized” from the bone marrow by applying a specific drug. As of 2022, data from DKMS indicates that peripheral extraction is used in 90% of stem cell donations collected from DKMS-registered donors, whereas bone marrow removal from the iliac crest (done through surgery under general anesthesia) is undertaken in the remaining 10% of collections.^3^ While DKMS rather prominently displayed information about stem cell extraction methods on its website^4^, our survey results reveal that a substantial fraction of registry members neither know the correct extraction methods nor their relative prevalence. Specifically, in our survey, we gave respondents the opportunity to choose among four possible stem cell extraction methods: peripheral (from the bloodstream), iliac crest (hip), spinal cord (spine), and cheek swab. These were elicited in a probabilistic manner; that is, we asked donors to indicate in what percentage of cases stem cells are obtained with each of these methods. We included these four potential methods because (a) “from the bloodstream” and “from the hip” are the methods actually used, (b) “spinal cord” and “cheek swab” are never used, (c) the “cheek swab” method is used to collect the initial sample at the time of recruitment, and thus registry members might incorrectly believe the donation method will be similar to the recruitment method, and (d) because in German language, the words for spinal cord (*Rückenmark*) and bone marrow (*Knochenmark*) could potentially be confused by registered donors.

Our results document, first, that incorrect beliefs about stem cell extraction methods are widely held throughout the donor population. As we also employ a measure of donor confidence in the point estimate of peripheral extraction, we can confirm that the beliefs become more accurate with increasing donor confidence. However, there is still a substantial fraction of donors who are confident and state a substantial likelihood of an unused method. Hence, our results document both a lack of knowledge (for underconfident donors) and strong misconceptions (in case of confident donors). Specifically, we find that only 45% of respondents in our representative survey stated that peripheral extraction is the most common extraction method. This method is much less invasive than extraction from the iliac crest, which involves a surgical procedure, and much less invasive than performing surgery on the donors’ spine (a method that is never used). Second, our findings indicate that registry members tend to retain their initial beliefs about the extraction method from the time of their registration and do not significantly update their understanding thereafter. This suggests that donors pay limited attention to new information provided after they have registered. Third, our results suggest that donors’ misconceptions about extraction methods might be an important driver in a donor’s decision to follow through with a stem-cell donation. Our analysis reveals that accurate beliefs regarding lower extraction costs are associated with a 2.2–2.9 percentage points higher willingness to donate, representing a 40% reduction in donor unavailability.

The paper proceeds as follows: Section "Methods" describes our survey and the data, and Sect. "Results" presents the results. Finally, Sect. "Beliefs and confidence in knowledge about extraction methods" concludes and offers implications of our findings for the management of stem-cell registries.

## Methods

To assess donors’ beliefs about stem cell extraction methods and the link to their willingness to donate, we designed a comprehensive survey. In October and November 2021, this survey was administered online (using the software LamaPoll) to individuals in Germany who had registered to potentially become stem cell donors. We partnered with DKMS, a leading international stem-cell donor registry, which has more than 7.7 million registered potential donors in Germany [13].

A total of 54,340 email invitations were sent out to registered donors who had neither donated stem cells nor received a confirmatory typing request before. The raw data contain 30,013 observations, with 6,386 cases with missing information on at least one of the main variables used in the empirical analyses. Hence, our final sample includes a total of 23,627 valid, i.e., non-missing, responses, which corresponds to a response rate of 43.5%. Among the survey respondents, 59.9% were female, 39.9% male, and less than 0.2% non-binary. The average age was 39 years (minimum: 19 years, maximum: 61 years). Hence, our sample of respondents is very similarly distributed as compared to the full population of potential donors registered with DKMS Germany (female: 59.2%, male: 40.8%, mean age: 39 years).

Our main outcome variable is a potential donor’s subjective probability that they would donate stem cells, should they be identified as a close match for a patient. Importantly, survey participants were asked about this probability before we elicited their beliefs about extraction methods. The probability is captured through the following survey item: “*When answering the questions below, please assume that you are able to donate stem cells—that is, you have the time, you live in Germany, and there are no medical reasons preventing you from donating. Imagine that you are contacted* [*i. within the next week; ii. in 3 months; iii. in a year; iv. in 5 years*] *to donate stem cells to a patient. What is the probability that you will actually donate?*”

In our analyses, we mainly focus on the sub-items “within the next week” and “in a year”, as we aim to capture donors’ intentions to donate close to the present and to measure their intentions in the near future. The average willingness to donate varies between 92.52% for a donation next week and 94.23% for a donation in a year; see Table 1. The Pearson correlation coefficient between these two variables is 0.669 $(p<0.001)$. Because our survey question asked prospective donors to exclude medical and other reasons for non-eligibility, this is one potential reason why the proportion is higher than actual CT availability rates for DKMS Germany, which are around 72% in 2023 [13]. Additionally, responses may be influenced by hypothetical scenarios and social desirability bias.

**Table 1:** Summary statistics. $N = 23,627$

Variables	Mean	S.D.	Min.	Max.	Med.
*Assessment of extraction methods and confidence*					
Extraction from cheek	7.35	14.39	0	100	0
Extraction from spine	23.02	25.23	0	100	15
Extraction from iliac crest (hip bone)	30.44	26.04	0	100	20
Extraction from bloodstream (peripheral)	39.18	30.23	0	100	35
Confidence in extraction via bloodstream	3.01	1.79	1	7	3
Correct belief	0.11	0.31	0	1	0
Correct belief with 10% tolerance	0.20	0.40	0	1	0
*Intention to donate*					
Donation next week	92.52	16.62	0	100	100
Donation in 3 months	94.03	14.25	0	100	100
Donation next year	94.23	13.46	0	100	100
Donation in 5 years	92.88	15.50	0	100	100
*Attitudes and preferences*					
Reciprocity	8.03	2.05	0	10	8
Trust	5.75	2.24	0	10	6
Procrastination	4.80	2.85	0	10	5
Time	7.29	1.64	0	10	7
Altruism	8.42	1.46	0	10	9
Risk	5.87	1.96	0	10	6
Health risk	4.57	2.20	0	10	5
*Additional covariates*					
General donation knowledge	3.21	1.11	1	5	3
Male	0.40	0.49	0	1	0
Female	0.60	0.49	0	1	1
Non-binary	0	0.04	0	1	0
Age	38.89	11.19	19	61	38
Donated money or volunteered	0.69	0.46	0	1	1
Donated blood	0.63	0.48	0	1	1
Time until registration: $<24$ hours	0.12	0.32	0	1	0
Time until registration: 1 day to 1 month	0.34	0.47	0	1	0
Time until registration: 1–6 months	0.18	0.38	0	1	0
Time until registration: 6–12 months	0.07	0.26	0	1	0
Time until registration: 1–2 years	0.11	0.31	0	1	0
Time until registration: more than 2 years	0.18	0.39	0	1	0

*Notes.* This table shows summary statistics of all variables used in our study. Quantitative belief items are described by extraction, which are mutually exclusive subjective probabilities of extraction from the respective method. confidence In extraction via bloodstream is the associated qualitative item measuring confidence in one’s response to the bloodstream survey item. The variable correct belief takes the value 1 if a donor gave the highest probability of extraction to periphery, the second highest to iliac crest, and zero probability to both spinal and cheek swab extraction. Errors in potential donors’ stated frequencies of up to 10 percentage points for each of spine and cheek extraction method is accounted for in the variable correct belief with 10% tolerance. Intention to donate is measured for four future points in time, and is a self-reported intention to follow-through with donation, given one is medically eligible to donate. Attitudes and preferences are measured using Falk et al. [6] for all items except health risk, which is from Dohmen et al. [4]. General donation knowledge measures how well donors can remember the overall donation process. Time until registration measures how long donors took to register as a stem cell donor, from the point in time they first heard of it

To investigate how informed potential donors are about the methods for extracting stem cells, we elicited donors’ beliefs through the following survey item: “*Based on your best estimate, in what percentage of cases are stem cells collected from the donor in the following ways?* [*i. Bone marrow from the iliac crest (hip); ii. From the spinal cord; iii. From the bloodstream; iv. By cheek swab*].”^5^ The predominant extraction method of stem cells is through the bloodstream (peripheral). We thus subsequently elicited donors’ confidence about their response to the peripheral extraction method survey item. The response categories ranged from 1 (not at all confident) to 7 (very confident). For the empirical analysis, we generate a categorical variable labeled “confident", which classifies donors as being *confident* at or above a 6 out of 7 scale points, *moderately confident* if they respond with 3, 4 or 5, and as *not confident* if they respond with a 1 or a 2. We further generate a variable called correct belief, our main independent variable, which captures whether respondents ranked extraction via the bloodstream and iliac crest in terms of their relative frequencies correctly (i.e., they ranked peripheral collection higher than aspiration from the iliac crest) *and* assigned zero probability to the never-used extraction methods from the spine and via cheek swab. The variable correct belief with 10% tolerance additionally allows donors who correctly ranked the iliac crest and blood extraction to state a frequency of up to 10 percentage points for each never-used method of spine or cheek extraction (i.e., we allow for a 10 percentage points tolerance level for each never-used method). A further important control variable is a potential donor’s knowledge about the stem cell donation process as a whole, which might also be a proxy for the level of interest of a registered donor in the topic of stem cell donation in general. The corresponding survey item reads as follows: “*Some time has passed since your registration. How precisely do you still know how stem cell donation works?*” Response categories ranged from 1 (*not precisely at all*) to 5 (*very precisely*) on a 5-point Likert scale, with a mean value of 3.2 (median: 3; see Fig. 8 in Appendix A). The level of confidence is positively correlated with the item measuring a potential donor’s knowledge about the donation process (Spearman rank test $\rho = 0.375$, $p<0.001$).

We also account for registry members’ characteristics that potentially relate to their stated likelihood that they would donate stem cells. To this end, we collected information about registry members’ demographics, including age and gender, as well as health-related risk attitudes, time preferences, general trust in others, altruism, positive reciprocity, and procrastination tendencies, using a battery of experimentally-validated survey items [4, 6, 7]. Further, we asked donors if they have ever donated blood, volunteered, or donated money to a charity. Table 7 in Appendix A lists and details all items we included in the survey. Descriptive statistics of all variables used in the empirical analyses are provided in Table 1.

## Results

### Beliefs and confidence in knowledge about extraction methods

This section explores to what extent individuals are informed about current stem cell extraction methods and whether beliefs about the extraction methods are moderated by their stated confidence in their own knowledge about extraction methods. In Sect. "Main sample: Registry members", we analyze a sample of donors registered with DKMS. We benchmark these findings with a random sample of non-registered individuals from the general population in Sect. "Comparison sample: General population".

#### Main sample: Registry members

We now analyze to what extent registry members are informed about current stem-cell extraction methods. As DKMS posts on their website, the average probability of extraction being peripheral (from the bloodstream) at the time of the survey was and still is approximately 90%. In the remaining 10% of the cases, bone marrow is collected using a puncture needle in the iliac crest of the pelvis (hip bone), which takes place under general anesthesia.^6^ Our survey results reveal, however, a surprisingly high level of misconception: Potential donors state that extractions occur, on average, in only 39.2% of the cases from the bloodstream, in 30.4% of the cases from the iliac crest, in 23% of the cases from the spine, and in 7.4% from cells gathered via a cheek swab; see Table 1.

**Fig. 1:**
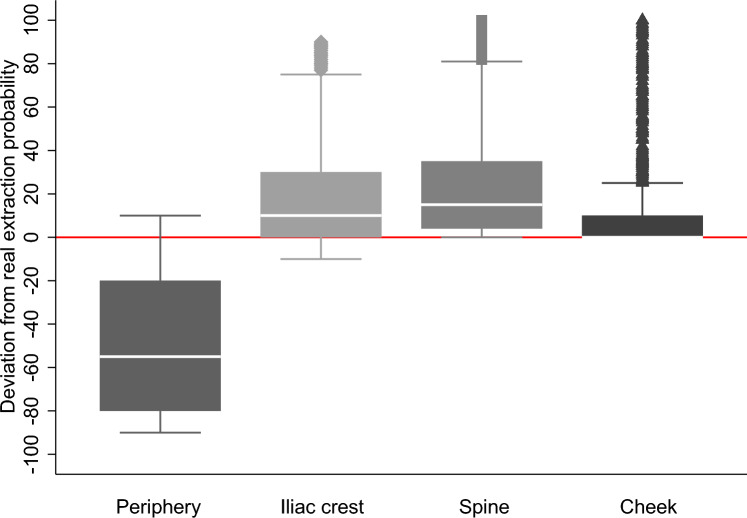
Deviations between survey responses and actual extraction methods (box plot with 95% confidence intervals). The actual proportions of extraction methods conducted at DKMS Germany are: peripheral 90%, iliac crest 10%, and spine as well as cheek swab 0%

Figure 1 emphasizes this misconception about extraction methods by illustrating the deviations between potential donors’ survey responses and actual frequencies of stem-cell extraction methods employed at DKMS Germany. It is also noteworthy that there is a difference by age, with older donors being more likely to report iliac-crest extraction relative to peripheral blood; see Fig. 6 in Appendix A.

Further analyses show that 80% of potential donors incorrectly allocate a positive probability to the two extraction methods, which are never used, namely spine and cheek swab. Even if we allow for a 10 percentage points tolerance level in stated probabilities to each unused extraction method to account for the fact that respondents might, for example, accidentally make erroneous choices, there are still 59.5% of respondents allocating probabilities higher than 10% to these never-used methods. Again, these results highlight a surprisingly high level of misconception among registered potential donors.

In terms of relative importance of extraction methods, we observe that only 45% of prospective donors allocated the highest percentage to extraction from the bloodstream. Conditional on assigning the highest probability to peripheral, 23% of donors correctly stated that extraction from the iliac crest is the second most likely method (which is only 10% of all cases). According to our generated variable correct belief, which captures whether respondents correctly indicated the ranking of the two common stem-cell extraction methods in terms of their relative frequencies and simultaneously assigned zero probability to the never-used extraction methods from the spine and via a cheek swab, our survey results reveal that only 11% of donors, on average, understood the possible extraction methods correctly. Also allowing for a 10 percentage points tolerance level in this definition, captured by the variable correct belief with 10% tolerance, still only 20% of donors understood the possible extraction methods correctly. Again, this confirms that a substantial fraction of donors is misinformed about the stem cell extraction methods although being registered.

In the following, we investigate whether potential donors’ beliefs about the extraction methods are moderated by their stated level of confidence in their responses to the peripheral extraction method survey item. In general, we find a low level of reported confidence among potential donors about this mostly-used extraction method. The distribution of responses is right-skewed, with a mean and median of 3 (out of 7) and nearly half of the respondents indicating not feeling confident at all (a 1 or a 2); see Fig. 7 in Appendix A.

**Fig. 2:**
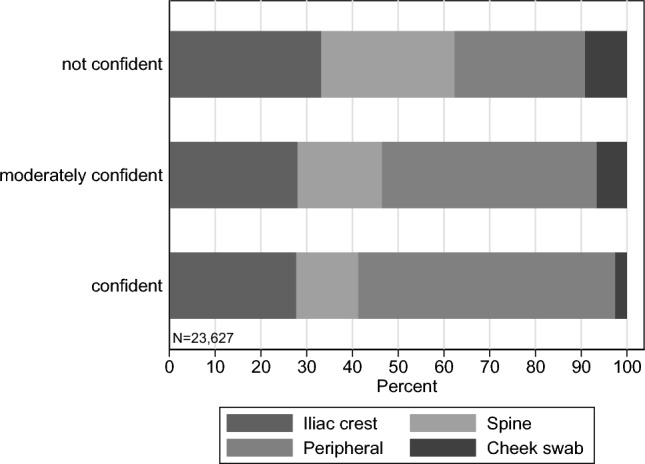
Average potential donors’ beliefs about extraction methods by confidence level. To categorize potential donors’ confidence we label “confident” for scale points 6–7 (*N* = 2531), “moderately confident” for 3–5 (*N* = 9,914), and “not confident” for 1–2 (*N* = 11,182). Distribution: Confident: 10.7%, moderately confident: 42.0%, not confident: 47.3% (*N* = 23,627)

Interestingly, the level of confidence explains substantial heterogeneity in the answers about the extraction methods. Figure 2, with extraction beliefs stratified by the three confidence levels (i.e., confident, moderately confident, and not confident), indicates a substantially higher share of donors who report more plausible percentage values for extraction methods when they feel more confident. Similarly, Fig. 9 in Appendix A shows that the fraction of potential donors that gave positive probabilities to the never-used extraction method via cheek swab or from the spine is non-negligible and is strongly decreasing as the respondents’ confidence level increases. But even when potential donors perceive their beliefs to be fairly accurate, they still attribute a significant fraction of stem cell extractions to be coming from the never-used methods of cheek swab or spine, again indicating substantial misconceptions among registered potential donors. Taken together, we find severe misconceptions about extraction methods among potential stem cell donor types.

This substantial heterogeneity in how the level of confidence influences answers about the extraction methods is confirmed in multivariate analyses shown in Table 2. As can be seen in Columns 1 to 4, the level of confidence explains between 1% and 12% of the overall variation in beliefs about extraction methods.

**Table 2:** Donor beliefs and donor confidence about extraction methods

	(1)	(2)	(3)	(4)	(5)
	Peripheral	Cheek swab	Spine	Iliac crest	Correct belief
Moderately confident	18.4572$$^{***}$$ (0.3910)	– 2.6074$$^{***}$$ (0.1965)	– 10.7351$$^{***}$$ (0.3379)	– 5.1147$$^{***}$$ (0.3574)	0.1196$$^{***}$$ (0.0041)
Confident	27.7282$$^{***}$$ (0.6239)	– 6.6509$$^{***}$$ (0.3135)	– 15.6744$$^{***}$$ (0.5392)	– 5.4029$$^{***}$$ (0.5703)	0.3098$$^{***}$$ (0.0066)
Reference group mean (not confident)	28.4668$$^{***}$$ (0.2680)	9.1571$$^{***}$$ (0.1347)	29.2076$$^{***}$$ (0.2317)	33.1684$$^{***}$$ (0.2450)	0.0276$$^{***}$$ (0.0028)
Adjusted $R^2$	0.12	0.02	0.06	0.01	0.09
Obs.	23,627	23,627	23,627	23,627	23,627

*Notes.* This table shows estimation results from OLS regressions. The dependent variables in Columns 1 to 4 are percentage point beliefs about extraction methods. In Column 5, “Correct belief” denotes a dummy variable that takes the value 1 if a respondent correctly stated the order of extraction method likelihood and stated zero probability to extraction from cheek and spine, and zero otherwise. For the categorization of the confidence variable, see Fig. 2. Robust standard errors are reported in parentheses. No controls included. The symbols *, **, and *** represent significance levels of 10%, 5%, and 1%, respectively

#### Comparison sample: General population

Registered stem cell donors self-selected into a stem cell registry and seem potentially better informed about stem cell collection methods compared to the general population. As a result, misperceptions about stem cell collection methods are probably even larger in the general population compared to our survey population. To investigate the role of self selection, we ran an online survey with a random sample of the German population in February 2025. The survey contains a subset of items from our baseline survey presented above. In detail, we partnered with OmniQuest, a leading German survey and market research company, to conduct an online survey (CAWI) with 500 randomly selected participants living in Germany. Our sample is representative of the German population with respect to age categories. Importantly, age is restricted to 18 to 55 years and participants aged 18 to 25 years were overrepresented when sending out invitations to make the population sample as similar as possible to our sample of registered donors in terms of age (where also 18-25 year old potential donors are overrepresented). To study the level of misinformation in the general population, we dropped survey respondents who are registered with a stem cell registry (124 observations) and those who have already donated stem cells (18 observations). Hence, the final sample contains 358 observations from individuals not registered with any stem cell registry. The average age in our population sample is 36.7 years and 50.3% are females.

**Fig. 3:**
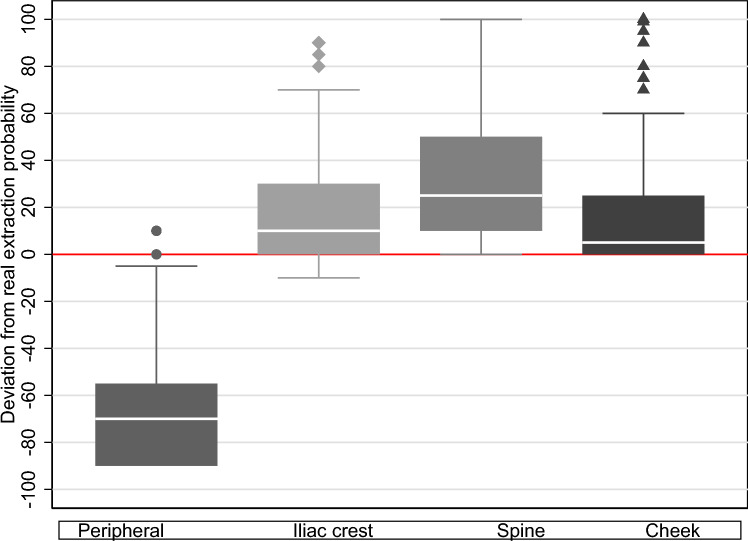
Deviations between survey responses and actual extraction methods for non-registered individuals from random population sample (box plot with 95% confidence intervals). The actual proportions of extraction methods conducted at DKMS Germany are: peripheral 90%, iliac crest 10%, and spine as well as cheek swab 0%

We find that misperceptions about stem cell collection methods are much larger in the general population compared to our survey sample of donors registered with the DKMS. In particular, survey respondents from the general population state that extractions occur, on average, in only 25% of the cases from the bloodstream (DKMS sample: 39.2%), in 25.8% of the cases from the iliac crest (DKMS sample: 30.4%), in 34% of the cases from the spine (DKMS sample: 23%), and in 15.1% from cells collected by a cheek swab (DKMS sample: 7.4%). Hence, beliefs in the general population about the relative proportion of two commonly used stem cell collection methods are smaller than those in the DKMS sample, whereas the importance of the two never-used collection methods is even greater. This larger misconceptions are supported by Fig. 3, which replicates Fig. 1 for the general population sample.

**Fig. 4:**
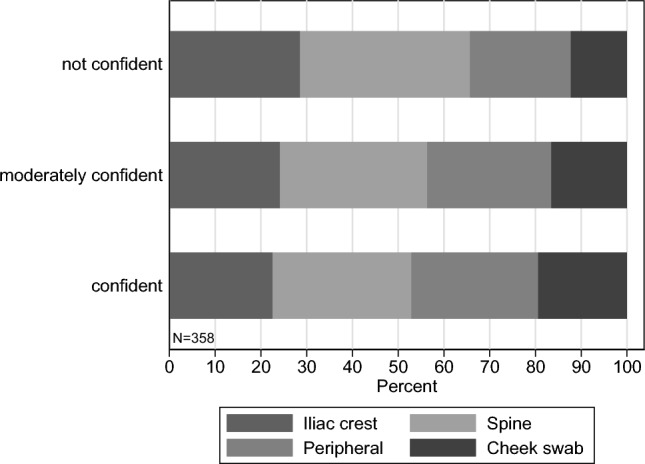
Average beliefs of random population sample about extraction methods by confidence level. To categorize potential donors’ confidence we label “confident” for scale points 6–7 (*N* = 62), “moderately confident” for 3–5 (*N* = 139), and “not confident” for 1–2 (*N* = 157). Distribution: Confident: 17.3%, moderately confident: 38.8%, not confident: 43.9% (*N* = 358)

Second, Fig. 4 replicates the analysis shown in Fig. 2, where we illustrate the average beliefs about the collection methods by confidence level for the sample of the general population. Again, even individuals in the general population who consider themselves confident in their response to the bloodstream item attribute a much higher importance to extraction from the spine as well as a much lower importance of collection via the bloodstream as compared to the DKMS sample. Strikingly, in the general population sample, the relative proportion of stem cell extraction via cheek swabs increases in the level of confidence, which is opposite to what we see in the DKMS sample.

Further, we analyze whether the misconceptions about extraction methods affect the likelihood of becoming a registered donor in the first place. We have integrated a survey item on the willingness to register with a stem cell registry, with the following response categories on a four-point Likert scale: 1 (never), 2 (rather not), 3 (eventually yes) and 4 (definitely yes). We create a dummy variable taking the value 1 in case an individual responds with 3 or 4, and 0, otherwise (mean: 0.54). We then estimate a probit regression (with marginal effects) with this dummy as the dependent variable and individuals’ responses about the relative frequency of stem cell collection methods as well as their confidence level as main independent variables, while controlling for individuals’ age, gender, and education. The results in Table 12 in Appendix A show that individuals from the general population, who state a higher confidence in their answer to the bloodstream extraction item, show a 7.6 percentage point higher likelihood of planning to join a stem cell registry (significant at the 1% level). Furthermore, assigning higher percentages to extraction via the iliac crest and higher education is positively correlated with the likelihood of registration.

To conclude, although registered donors are better informed about stem cell collection methods compared to a random draw of the population, we still find a substantial level of misconception among registry members.

### Variation over time in potential donors’ beliefs about extraction methods

Our results so far lead to an important question: Why do we observe such a large heterogeneity in the beliefs of registered donors about extraction methods? To investigate this, we first analyze the beliefs by age groups. Donor age is a crucial parameter for stem cell donation, since transplant physicians prefer young male donors, who should ideally register at age 17 or 18. Also, the relative importance of peripheral stem cell collections (as compared to bone marrow removal from the iliac crest) increased over time; see Fig. 10 in Appendix A. After the introduction of peripheral donations in the mid-1990s, they quickly reached a share of around 80%, which then remained constant for almost two decades until there was a jump in 2020 due to the pandemic (mainly due to logistical advantages over bone marrow removal). Since older registry members are, on average, likely to be longer in the registry than, for example, 20-year-olds, their knowledge over current extraction methods is potentially not as precise and up-to-date, as information is likely provided upon registration. The reason is that relative frequencies of extraction methods have substantially changed over time, and donors may potentially not keep up to date with these changes in extraction methods after initially agreeing to donate under the circumstances of donation at the time of their registration.

**Fig. 5:**
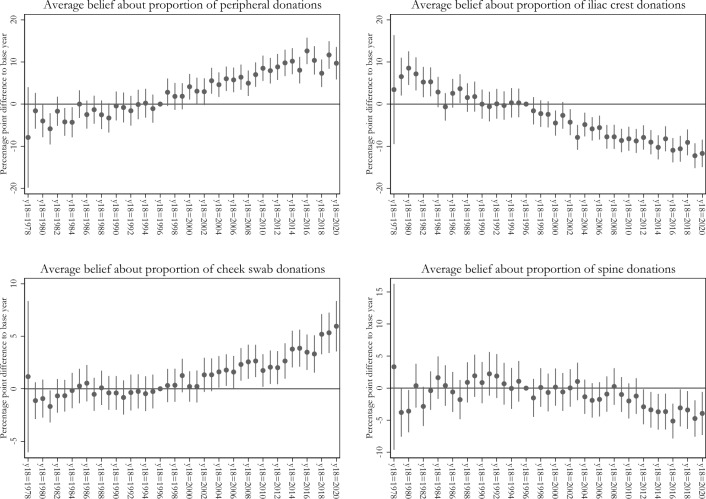
The left figure shows regression results of percentage point beliefs of peripheral extraction on the year donors turned 18 as the main explanatory variable (1996 = base year). The top-right figure shows the same regression with iliac crest extraction as the outcome, bottom-left for cheek swab donations and bottom-right for spine donations. Controls include preferences, previous blood donation, previous volunteering or monetary donation, time until registration, and gender. 95% robust confidence intervals shown as whiskers

**Fig. 6:**
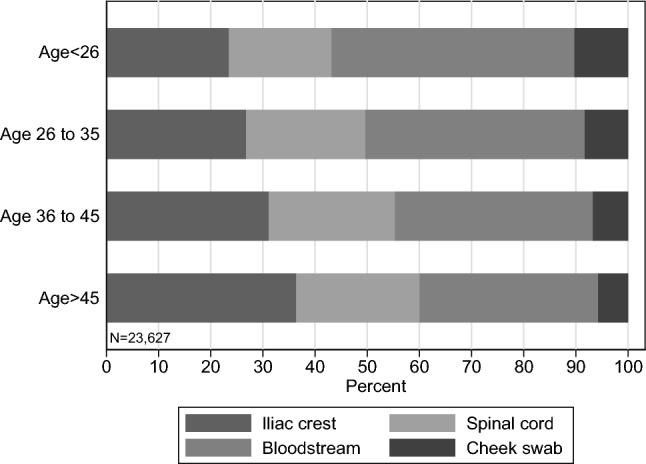
Average prospective donors’ beliefs about extraction methods by age. $N = 23,627$

**Fig. 7:**
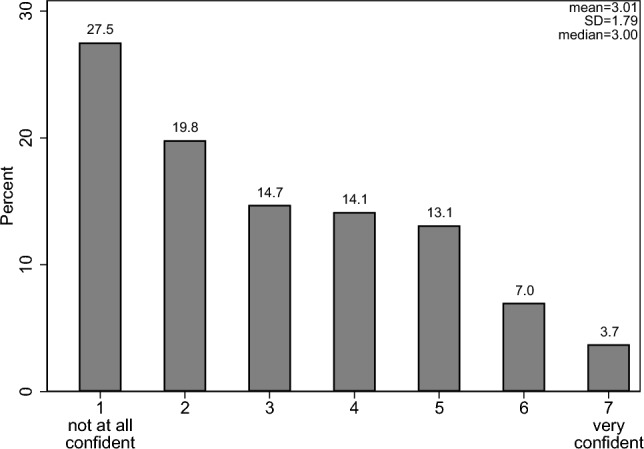
Confidence about peripheral as an extraction method. $N = 23,627$

**Fig. 8:**
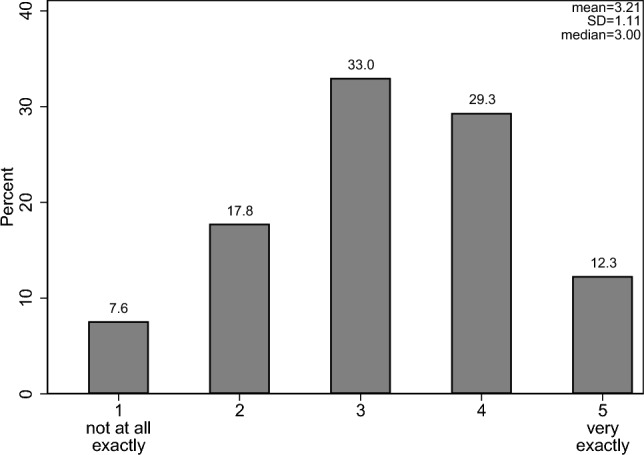
Knowledge about the overall donation process. $N = 23,627$

**Fig. 9:**
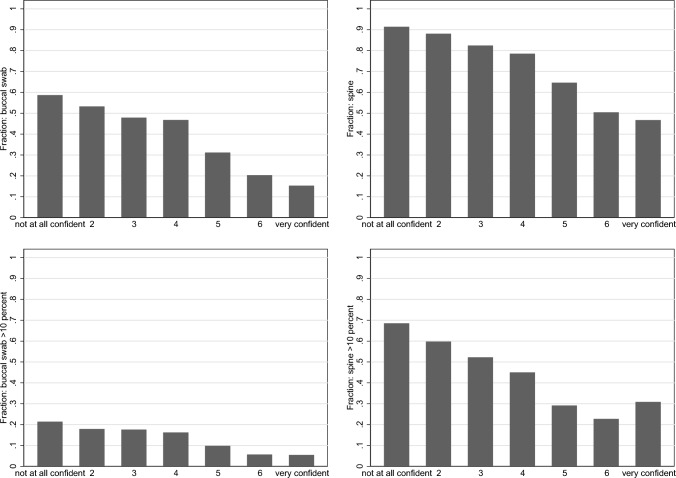
Fraction of prospective donors that believe stem cell donations are extracted using the never-used methods cheek swab (top-left and bottom-left) and spine (top-right and bottom-right) by confidence level. Definition: Confident: Scale points 6-7 $N = 2,531$; moderately confident: 3–5 $N = 9,914$, not confident: 1–2 $N = 11,182$. Distribution: Confident: 10.7%, moderately confident: 42.0%, not confident: 47.3%. $N = 23,627$

**Fig. 10:**
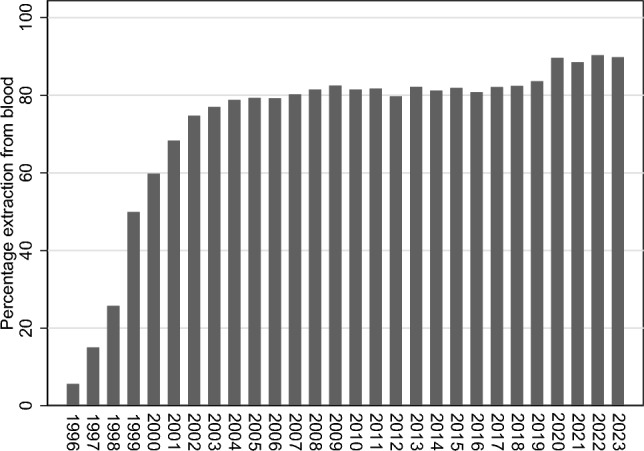
Fraction of peripheral extractions by year. Source: DKMS Germany

To test this hypothesis, we created a variable that reflects the frequency of peripheral extraction when the donor surveyed was 18 years old—in particular, when donors became eligible to donate. Note that we do not have information on the exact year of registration of our survey participants. But in the full sample of DKMS Germany, about 30% of all currently registered donors have registered at the age of 18 to 22 years, which supports the use of our proxy variable. If donors only inform themselves about extraction methods when entering the registry (e.g., at a donor drive) and do not update their beliefs thereafter, then we should see that donors’ beliefs are more likely to be correct if they became 18 years old around the time of, and persistently after, the increase in the peripheral extraction method. Indeed, in Fig. 5, we observe an increase in beliefs about peripheral stem-cell collection for donors who turned 18 in 1998, where about 25% of extractions were peripheral, which continues to increase until well after 2010. Further, when using beliefs about iliac-crest donations as the outcome, we observe a decline in beliefs in the younger cohorts. This is consistent with our interpretation.

We observe an increase in beliefs that a cheek swab can be used to donate. This misconception potentially arises from the point of registration, where donors nowadays use a cheek swab to collect a saliva sample that is used to for HLA-typing. It is unclear whether donors think they have already donated by giving a saliva sample. One advertising slogan says: “*Mund auf. Stäbchen rein. Spender sein*” (Insert a cheek swab, be a donor).^7^ This might potentially be misleading as one does not get adequate information about how extractions work.

On the other hand, it appears that younger cohorts of registered donors are less likely to believe a stem-cell donation comes from the spinal cord. This is potentially because donation has overall become less invasive in the form of peripheral donations, and hence, less rumors about invasive procedures spread. Of course, this is only one potential channel. Also, it is likely that donors who are *inattentive* believe that the cheek swab is the method used.

### Beliefs about extraction method and intention to donate

Next, we investigate the link between donors’ beliefs about the extraction method and their willingness to donate stem cells. To this end, we correlate the variable correct belief with the self-reported likelihood (in percentage points) that one would actually donate stem cells if (being medically eligible and) asked next week or in a year. As before, we control for knowledge about the donation process, economic preferences [4, 6], any previous blood donation, volunteering and monetary donations, and age by gender interactions in our analyses.

Table 3 shows regression results for the willingness to donate within the next week (Columns 1 to 3) and within the next year (Columns 4 to 6) as the dependent variable. Having correctly ranked beliefs about the extraction methods is associated with a 2.9 percentage point (pp) higher willingness to donate; see Column 1 of Table 3. Recall that the unconditional baseline availability is 92.5%, therefore reducing stated donor attrition by 38.7% (2.9/7.5).^8^ The corresponding estimated coefficient when the dependent variable is the respondent’s willingness to donate within the next year is only slightly smaller at 2.2pp (Column 4). Additionally, accounting for donors’ knowledge about the stem-cell donation process and their confidence about the blood extraction method yields very similar estimates; see Columns 2 and 5 of Table 3. In Columns 3 and 6, we also include interaction terms between the level of confidence about extraction methods and our proxies for correct beliefs, as well as donation knowledge and beliefs. The interaction terms are not statistically significant in three out of four cases, indicating that the positive association between correct beliefs and the willingness to donate does not largely depend on the donors’ confidence about the peripheral extraction method. We also include economic preference measures, which do not change the main findings either, though being themselves correlated with donation intentions in the expected direction.^9^

We conduct several checks to probe the robustness of our results. First, these findings are robust when we allow survey participants to make errors when stating the probabilities on the extraction methods; see Table 6 in Appendix A. Second, using intentions to donate in three months and in five years as alternative outcome variables yield very similar results; see Table 10 in the Appendix. Third, using a fractional probit regression, reporting average marginal effects in Table 9 in the Appendix, with the outcome variable re-scaled between 0 and 1, shows our main results are robust and very similar in magnitude.

Overall, these results confirm our interpretation that potential donors’ misconceptions about stem cell collection methods—particularly about methods that are never used—can have detrimental effects on the willingness to follow through with a donation. The results suggest a mechanism by which registry members who are better informed about extraction methods are more likely to have lower perceived donation costs and, hence, are more likely to intend to donate.

**Table 3:** Correct belief about extraction methods and follow-through probability

Dep. variable:	Intention to donate next week			Intention to donate next year		
	(1)	(2)	(3)	(4)	(5)	(6)
Correct belief	2.882$$^{***}$$ (0.292)	1.832$$^{***}$$ (0.313)	2.305$$^{***}$$ (0.410)	2.208$$^{***}$$ (0.226)	1.532$$^{***}$$ (0.244)	1.774$$^{***}$$ (0.304)
Donation knowledge (std.)		1.442$$^{***}$$ (0.122)	1.326$$^{***}$$ (0.129)		1.111$$^{***}$$ (0.098)	0.981$$^{***}$$ (0.103)
Correct belief $\times$ donation knowledge (std.)			− 0.704$^{*}$ (0.361)			− 0.311 (0.309)
Extract blood certainty (std.)		0.107 (0.123)	0.112 (0.130)		− 0.043 (0.101)	− 0.044 (0.107)
Correct belief × extract blood certainty (std.)			− 0.011 (0.361)			0.016 (0.288)
Reciprocity (std.)			0.327$$^{***}$$ (0.111)			0.335$$^{***}$$ (0.091)
Trust (std.)			− 0.026 (0.110)			− 0.128 (0.088)
Time (std.)			0.200$$^{*}$$ (0.115)			0.195$$^{**}$$ (0.094)
Altruism (std.)			1.605$$^{***}$$ (0.126)			1.392$$^{***}$$ (0.103)
Health risk (std.)			0.495$$^{***}$$ (0.114)			0.483$$^{***}$$ (0.093)
Controls	Yes	Yes	Yes	Yes	Yes	Yes
Adjusted $R^2$	0.01	0.01	0.02	0.01	0.01	0.03
Observations	23,627	23,627	23,627	23,627	23,627	23,627

*Notes.* This table shows estimation results from OLS regressions. The dependent variable is the self-reported percentage point probability of being available to donate stem cells if asked within the next week (Columns 1 to 3), and in one year from being asked (Columns 3 to 6). “Correct belief” is a dummy variable that takes the value 1 if a respondent correctly stated the order of extraction method likelihood and stated zero probability to extraction from cheek and spine, and zero otherwise. Robust standard errors are reported in parentheses. Controls include all preferences (except general risk), previous blood donation dummy, previous volunteering or monetary donation dummy, time until registration, and age × gender. Measures used are reported in Appendix Table 7. “std.” means a variable is standardized with mean zero and unit variance. The symbols *, **, and *** represent significance levels of 10%, 5%, and 1%, respectively

**Table 4:** Extraction method estimates and follow-through probability

Dep. variable:	Intention to donate next week				Intention to donate next year			
	(1)	(2)	(3)	(4)	(5)	(6)	(7)	(8)
Extract iliac crest	0.0117$$^{***}$$(0.0041)				0.0061$$^{*}$$ (0.0033)			
Extract spine		− 0.0293$$^{***}$$(0.0046)				− 0.0160$$^{***}$$(0.0036)		
Extract blood			0.0272$$^{***}$$(0.0038)				0.0174$$^{***}$$(0.0031)	
Extract cheek				− 0.0543$$^{***}$$(0.0084)				− 0.0380$$^{***}$$(0.0066)
Controls	Yes	Yes	Yes	Yes	Yes	Yes	Yes	Yes
Adjusted $R^2$	0.02	0.03	0.03	0.03	0.02	0.03	0.03	0.03
Observations	23,627	23,627	23,627	23,627	23,627	23,627	23,627	23,627

*Notes.* Results from OLS regressions. The dependent variable is the self-reported percentage point probability of being available to donate stem cells if asked within the next week (cols. 1–4) and next year (cols. 5–8). Explanatory variables of interest are percentage point beliefs about the average usage of each extraction method. Robust standard errors are reported in parentheses. The symbols *,**, and *** represent significance levels of 10%, 5%, and 1%, respectively. Controls include donation knowledge, extract blood certainty, all preferences except general risk, previous blood donation dummy, previous volunteering or monetary donation dummy, time until registration, and age × gender

### Misconceptions and expected donation cost

From a policy perspective, it seems relevant to assess whether registry members have ever received correct information about how extraction is undertaken from a proper source of information, such as a registry, and whether this corresponds with more donations. One could argue that the relevant margin to assess this is whether potential donors believe mainly in an extraction method that is used at all (i.e., the extensive margin). This can be supported by the evidence we presented, indicating that respondents likely anchor their beliefs to the extraction method mainly used when they registered.

In the following, we further explore the nexus between misconceptions and perceived donation costs. We consider donors to be *informed* (*misinformed*) if the respondent states that peripheral or iliac crest extraction (cheek or spinal extraction) is used with more than a $50\%$ probability. Compared to our definition of being correctly informed (variable correct belief) used in baseline Appendix Table 3, the definition we apply here is, hence, less strict.^10^ Additionally, we separate donors along a cost dimension, as iliac crest and spinal extraction would be arguably more invasive than blood or cheek swab donation. Both low-cost and high-cost types contain one correct and one incorrect method each, allowing us to vary the cost within the informed and uninformed groups, respectively. Only 16.97% of donors believe that no method is used with more than 50% probability and, hence, are excluded from this analysis. 3.54% of donors believe cheek is mainly used, 40.47% peripheral, 16.40% spine, and 22.63% iliac crest.

We regress donation willingness (combining within the next week and next year together) on our high-cost method dummy for both the groups of informed and misinformed donors and low- and high-cost types in a stacked regression design. We start by reviewing the results for the groups of informed and misinformed donors presented in Columns (1) to (4) of Table 5. Donors who believe a high-cost method is mainly used are, on average, 0.45pp less likely to intend to follow through if they were informed about the right donation methods (Column (1)). On the other hand, misinformed donors do not significantly differ in their follow-through intentions with respect to cost (Column (3)). This adds doubt to comparability of informed and misinformed donors, and suggests baseline differences in their motivations to donate irrespective of their beliefs. The results remain quantitatively similar when including controls in Columns (2) and (4).

We next regress follow-through intentions on the informed dummy for each of the low-cost and high-cost method samples. Our results in Columns (5) and (7) of Table 5 show that being informed corresponds to a 2.22pp higher intention for the low-cost group and about 1.46pp for the high-cost group. Here, we suggest that this is largely a selection effect, as the misinformed and informed groups do not respond comparably when it comes to believing in a low- or high-cost method. Again, including our control vector does not change the results, as can be seen in Columns (6) and (8).

**Table 5:** Extraction method estimates and follow-through probability

Dep. variable: Sample	Intention to donate							
	Informed	Informed	Misinformed	Misinformed	Low cost	Low cost	High cost	High cost
	(1)	(2)	(3)	(4)	(5)	(6)	(7)	(8)
High-cost method	− 0.4492$$^{***}$$(0.1717)	− 0.5123$$^{***}$$(0.1866)	0.3137 (0.4415)	0.4839 (0.4408)				
Informed					2.2249$$^{***}$$(0.4134)	1.9645$$^{***}$$(0.4236)	1.4620$$^{***}$$(0.2316)	1.1972$$^{***}$$(0.2342)
Controls	No	Yes	No	Yes	No	Yes	No	Yes
Adjusted $R^2$	0.00	0.02	0.00	0.03	0.00	0.02	0.01	0.03
Obs.	29,816	29,816	9,420	9,420	20,794	20,794	18,442	18,442

*Notes.* Results from stacked OLS regressions. The dependent variable is the self-reported percentage point probability of being available to donate stem cells if asked within the next week and next year together in one regression in a stacked design with two constants estimated. Explanatory variables of interest are indicators of whether the respondent indicated to mainly believe extraction is done from a high-cost method (in informed vs. misinformed samples) or whether the method mainly suggested is correct (in the low cost and high cost samples). Controls include donation knowledge, extract blood certainty, all preferences except general risk, previous blood donation dummy, previous volunteering or monetary donation dummy, time until registration, and age × gender. The symbols *, **, and *** represent significance levels of 10%, 5%, and 1%, respectively

## Discussion

Our study showed that incorrect beliefs about stem-cell extraction methods are widely prevalent among registered potential donors in a major European stem cell registry. Even potential donors who are most confident about extraction methods have wrongful beliefs. Our results thus document both a lack of knowledge (for unconfident donors) and strong misconceptions (in the case of more confident donors). Second, our results suggest that registered potential donors anchor their beliefs about the extraction method to when they registered and do not substantially update their beliefs in the meantime. This indicates a limited attention to the information available after registration. Finally, we find that having correctly ranked beliefs about extraction methods is robustly and significantly associated with a higher stated willingness to donate stem cells in the near future.

Our results yield several implications for the management of stem cell registries. In case of a CT request, a case manager contacts the donor by phone to provide detailed information about both stem cell collection methods and to confirm the donor’s availability (for either method). Additionally, the donor’s contact details are updated and arrangements are made to send a blood draw kit. The donor is then advised to schedule an appointment with their primary care provider to complete the blood draw. Hence, this conversation at the time of a CT request might be a chance to correct registered donors’ misperceptions about stem cell collection methods.

On the downside, individuals’ misperceptions may be difficult to change at this step so close to the actual donation decision. This is especially important given that our results are strongest for a donation at a closer point in time. If individual beliefs are sticky—in particular, if they react slowly or not at all given new information, it may be critical to invest in information campaigns already prior to registration to correctly inform members of the registry, so that potential donors have time to adjust their beliefs and reconsider the costs and benefits of donation, before actually being asked to do so. There are other reasons why informing donors can have positive effects. For instance, engagement with the registry may increase (e.g., [10]) or they may be more likely to tell others how painless and convenient donation normally is. In addition, correct information on stem cell extraction methods can lower the *ex ante* psychological costs of joining the registry. Information provided may be most effective to causally change individuals’ donation behavior close to the actual donation stage. This is suggested by Elias et al.’s [5] experiment which assesses the effect of providing information on peoples’ attitudes toward paying kidney donors.

Lastly, our study has limitations. Although we document strong correlations between the level of information on stem cell collection methods and the stated willingness to donate in a large online survey, more research should analyze actual CT availability data to provide stronger causal evidence to support our main findings.

## A Additional tables and figures

See below Appendix Figs. 6, 7, 8, 9, 10 and Appendix Tables 6, 7, 8, 9, 10, 11 and 12 here.

**Table 6:** Correct information about extraction methods and follow-through probability: Correct belief with 10% tolerance

Dep. variable:	Intention to donate next week			Intention to donate next year		
	(1)	(2)	(3)	(4)	(5)	(6)
Correct belief with 10% tolerance	2.539$$^{***}$$ (0.238)	1.761$$^{***}$$ (0.260)	1.749$$^{***}$$ (0.296)	1.594$$^{***}$$ (0.199)	1.052$$^{***}$$ (0.218)	0.946$$^{***}$$ (0.251)
Donation knowledge (std.)		1.432$$^{***}$$ (0.122)	1.293$$^{***}$$ (0.135)		1.118$$^{***}$$ (0.099)	0.962$$^{***}$$ (0.107)
Extract blood certainty (std.)		0.060 (0.124)	-0.033 (0.140)		-0.031 (0.103)	-0.134 (0.115)
Correct 10% tolerance $\times$ extract blood certainty (std.)			0.455 (0.291)			0.482$^{*}$ (0.252)
Correct 10% tolerance $\times$ donation knowledge (std.)			-0.251 (0.300)			-0.029 (0.262)
Reciprocity (std.)			0.317$$^{***}$$ (0.111)			0.330$$^{***}$$ (0.091)
Trust (std.)			− 0.026 (0.109)			− 0.128 (0.088)
Time (std.)			0.203$$^{*}$$ (0.115)			0.200$$^{**}$$ (0.094)
Altruism (std.)			1.614$$^{***}$$ (0.126)			1.396$$^{***}$$ (0.103)
Health risk (std.)			0.512$$^{***}$$ (0.114)			0.485$$^{***}$$ (0.093)
Controls	Yes	Yes	Yes	Yes	Yes	Yes
Adjusted $R^2$	0.01	0.01	0.02	0.01	0.01	0.03
Observations	23,627	23,627	23,627	23,627	23,627	23,627

*Notes.* This table shows estimation results from OLS regressions. The dependent variable is the self-reported percentage point probability of being available to donate stem cells if asked within the next week. (Columns 1 to 3), and in 1 year from being asked (Columns 3 to 6). “Correct belief with 10% tolerance” is a dummy variable that takes the value of one if a respondent correctly stated the order of extraction method likelihood and stated at most a 10% probability to extraction from cheek or spine, and zero otherwise. Robust standard errors are reported in parentheses. The symbols *, **, and *** represent significance levels of 10%, 5%, and 1%, respectively. Controls include previous blood donation dummy, previous volunteering or monetary donation dummy, time until registration, and age$\times$gender. Measures used are reported in Table 7. “std.” means a variable is standardized with mean zero and unit variance

**Table 7:** Survey items

Survey item	Exact wording of item(s)	Scale
Donation follow-through	Imagine that you will be contacted in (A:5 years, B: one year, C: 3 months, D: one week). What is the likelihood that you will complete the donation?	Percentage
Stem cell collection beliefs	Based on your best estimate, in what percentage of cases are stem cells collected from the donor in the following ways? 1. bone marrow from the iliac crest (hip) 2. from the spinal cord 3. from the bloodstream 4. by cheek swab	Assign 100 percentage points in total to all items
Confidence in stem cell collection beliefs	You stated that, for example, there is a [$X\%$] chance that stem cells will be taken from the donor’s bloodstream. How sure are you about this answer you just gave?	7-point scale
Knowledge of donation process	Some time has passed since your registration. How exactly do you still know how a stem cell donation works?	5-point scale
Blood donor	Have you ever made a whole blood or plasma donation?	Yes, No, ineligible, prefer not to say
Volunteer	Have you given money or performed volunteering services to charity in the last 12 months?	Yes/No
Health-related risk (Dohmen 2011)	Please tell me, in general, how willing or unwilling you are to take health-related risks.	11-point
Time (Falk et al. 2018 GPS)	How willing are you to give up something that is beneficial for you today in order to benefit more from that in the future?	11-point
Altruism (Falk et al. 2018 GPS)	How willing are you to give to good causes without expecting anything in return?	11-point
Positive reciprocity (Falk et al. 2018 GPS)	When someone does me a favor, I am willing to return it.	11-point
Trust (Falk et al. 2018 GPS)	I assume that people have only the best intentions.	11-point
Procrastination (Falk et al. 2018 GPS)	C. I tend to postpone tasks even if I know it would be better to do them right away.	11-point
Age	How old are you?	In years
Gender	What is your gender?	Male/female/other

**Table 8:** Deviation from correct beliefs about extraction probabilities and intention to donate: heterogeneity by direction

Dep. variable:	Intention to donate next week				Intention to donate next year			
	(1)	(2)	(3)	(4)	(5)	(6)	(7)	(8)
Extract iliac crest$<=10$ $\times$ extract iliac crest difference	0.0583 (0.0440)				0.0558 (0.0349)			
Extract iliac crest$$>10$$ $$\times$$ extract iliac crest difference	0.0151$$^{***}$$(0.0046)				0.0091$$^{**}$$(0.0037)			
Extract blood$$<=90$$ $$\times$$ extract blood difference		-0.0255$$^{***}$$(0.0040)				-0.0168$$^{***}$$(0.0032)		
Extract blood$$>90$$ $$\times$$ extract blood difference		0.1923$$^{**}$$(0.0870)				0.0710 (0.0874)		
Extract Iliac crest$<=20$ $\times$ extract iliac crest difference			0.0219 (0.0189)				0.0230 (0.0149)	
Extract Iliac crest$$>20$$ $$\times$$ extract iliac crest difference			0.0180$$^{***}$$(0.0053)				0.0116$$^{***}$$(0.0042)	
Extract blood$$<=80$$ $$\times$$ extract blood difference				-0.0227$$^{***}$$(0.0042)				-0.0157$$^{***}$$(0.0034)
Extract blood$$>80$$ $$\times$$ extract blood difference				0.1156$$^{***}$$(0.0322)				0.0503$$^{*}$$(0.0297)
Controls	Yes	Yes	Yes	Yes	Yes	Yes	Yes	Yes
Adjusted $R^2$	0.02	0.03	0.02	0.03	0.02	0.03	0.02	0.03
Observations	23,627	23,627	23,627	23,627	23,627	23,627	23,627	23,627

*Notes.* Results from OLS regressions. The dependent variable is the self-reported percentage point probability of being available to donate stem cells if asked within the next week. Explanatory variables of interest are the percentage point differences between beliefs about the average usage of each extraction method and the true value communicated by the stem cell registry (either 10pp pelvic/90pp blood, or 20/80, since this has changed in recent years). Controls include donation knowledge, extract blood certainty, preferences, previous blood donation dummy, previous volunteering or monetary donation dummy, time until registration, and age$\times$gender. Robust standard errors are reported in parentheses. The symbols *,**, and *** represent significance levels of 10%, 5%, and 1%, respectively

**Table 9:** Fractional probit regression average marginal effects: Correct information about extraction methods and follow-through probability

Dep. variable:	Intention to donate next week			Intention to donate next year		
	(1)	(2)	(3)	(4)	(5)	(6)
Correct belief	0.0287$$^{***}$$ (0.0029)	0.0200$$^{***}$$ (0.0034)	0.0221$$^{***}$$ (0.0042)	0.0221$$^{***}$$ (0.0023)	0.0165$$^{***}$$ (0.0026)	0.0169$$^{***}$$ (0.0031)
Extract blood certainty (std.)		0.0013 (0.0013)	0.0013 (0.0013)		− 0.0003 (0.0010)	− 0.0003 (0.0010)
Donation knowledge (std.)			0.0124$$^{***}$$ (0.0012)			0.0094$$^{***}$$ (0.0010)
Controls	Yes	Yes	Yes	Yes	Yes	Yes
Observations	23,627	23,627	23,627	23,627	23,627	23,627

*Notes.* This table shows estimation results from fractional probit regressions (average marginal effects. The dependent variable is the self-reported probability (re-scaled to be zero to one) of being available to donate stem cells if asked within the next week. (Columns 1 to 3), and in 1 year from being asked (Columns 3 to 6). “Correct belief” is a dummy variable that takes the value 1 if a respondent correctly stated the order of extraction method likelihood and stated zero probability to extraction from cheek or spine, and zero otherwise. Robust standard errors are reported in parentheses. The symbols *, **, and *** represent significance levels of 10%, 5%, and 1%, respectively. Controls include preferences, previous blood donation dummy, previous volunteering or monetary donation dummy, time until registration, and age × gender. Measures used are reported in Table 7

**Table 10:** Correct information about extraction methods and follow-through probability

Dep. variable:	Intention to donate in 3 months			Intention to donate in 5 years		
	(1)	(2)	(3)	(4)	(5)	(6)
Correct belief	2.459$$^{***}$$ (0.232)	1.602$$^{***}$$ (0.251)	1.780$$^{***}$$ (0.323)	2.237$$^{***}$$ (0.273)	1.607$$^{***}$$ (0.293)	2.026$$^{***}$$ (0.359)
Donation knowledge (std.)		1.205$$^{***}$$ (0.105)	1.085$$^{***}$$ (0.110)		1.093$$^{***}$$ (0.110)	0.931$$^{***}$$ (0.116)
Extract blood certainty (std.)		0.070 (0.105)	0.045 (0.112)		-0.075 (0.116)	-0.037 (0.124)
Correct belief $\times$ extract blood certainty (std.)			0.258 (0.295)			-0.389 (0.318)
Correct belief $\times$ donation knowledge (std.)			-0.532$^{*}$ (0.305)			-0.034 (0.346)
Reciprocity (std.)			0.293$$^{***}$$ (0.096)			0.341$$^{***}$$ (0.107)
Trust (std.)			-0.045 (0.094)			-0.030 (0.104)
Time (std.)			0.179$$^{*}$$ (0.100)			0.342$$^{***}$$ (0.111)
Altruism (std.)			1.419$$^{***}$$ (0.107)			1.416$$^{***}$$ (0.116)
Health risk (std.)			0.569$$^{***}$$ (0.099)			0.457$$^{***}$$ (0.107)
Controls	Yes	Yes	Yes	Yes	Yes	Yes
Adjusted $R^2$	0.01	0.01	0.03	0.01	0.02	0.03
Observations	23,627	23,627	23,627	23,627	23,627	23,627

*Notes.* This table shows estimation results from OLS regressions. The dependent variable is the self-reported percentage point probability of being available to donate stem cells if asked in three months. (Columns 1 to 3), and in 5 years from being asked (Columns 3 to 6). “Correct belief” is a dummy variable that takes the value 1 if a respondent correctly stated the order of extraction method likelihood and stated zero probability to extraction from cheek or spine, and zero otherwise. Robust standard errors are reported in parentheses. The symbols *, **, and *** represent significance levels of 10%, 5%, and 1%, respectively. Controls include preferences, previous blood donation dummy, previous volunteering or monetary donation dummy, time until registration, and age$\times$gender. Measures used are reported in Table 7. “std.” means a variable is standardized with mean zero and unit variance

**Table 11:** Extraction method estimates and follow-through probability: robustness above 60% beliefs

Dep. variable: Sample	Intention to donate							
	Informed	Informed	Misinformed	Misinformed	Low cost	Low cost	High cost	High cost
	(1)	(2)	(3)	(4)	(5)	(6)	(7)	(8)
High-cost method	− 0.3512$^{*}$(0.1978)	− 0.3975$^{*}$(0.2151)	− 0.3950 (0.5346)	− 0.1318 (0.5414)				
Informed					1.7483$$^{***}$$(0.4988)	1.6139$$^{***}$$ (0.5197)	1.7921$$^{***}$$(0.2759)	1.4157$$^{***}$$(0.2774)
Controls	No	Yes	No	Yes	No	Yes	No	Yes
Adjusted $R^2$	0.00	0.02	0.01	0.03	0.00	0.02	0.01	0.03
Obs.	22,908	22,908	6,472	6,472	16,810	16,810	12,570	12,570

*Notes.* Results from stacked OLS regressions. The dependent variable is the self-reported percentage point probability of being available to donate stem cells if asked within the next week and next year together in one regression in a stacked design with two constants estimated. Explanatory variables of interest are indicators of whether the respondent indicated to mainly (above 60% probability) believe extraction is done from a high-cost method (in informed vs. misinformed samples) or whether the method mainly (above 60% probability) suggested is correct (in the low cost and high cost samples). The symbols *, **, and *** represent significance levels of 10%, 5%, and 1%, respectively. Controls include donation knowledge, extract blood certainty, all preferences except general risk, previous blood donation dummy, previous volunteering or monetary donation dummy, time until registration, and age$\times$gender

**Table 12:** General population: Willingness to register

Dep. variable:	Willingness to register (0/1)
Extract cheek	0.0014 (0.0016)
Extract iliac crest	0.0038$$^{***}$$ (0.0014)
Extract spine	0.0012 (0.0012)
Confident	0.0759$$^{***}$$ (0.0144)
Individual controls	Yes
Pseudo $R^2$	0.15
Obs.	358

*Notes.* This table shows estimation results from probit regressions. Marginal effects reported. The dependent variable is the willingness to register with a stem cell registry (0/1). Reference group: extraction via the bloodstream. Robust standard errors are reported in parentheses. The symbols *, **, and *** represent significance levels of 10%, 5%, and 1%, respectively

## Footnotes

1 At DKMS Germany, a major stem cell donor registry, the attrition rate (i.e., the percentage of donors who do not proceed with a donation after they are matched to a patient) was 28% in 2023 [13]. Though substantial, the attrition rate in Germany is lower as compared to, for instance, the US, with estimates of 36% [8], 40% [15], 47% [12], 50% [14] and most recently even 70.6% [13].

2 See, for example, https://www.healthing.ca/wellness/myths-around-stem-cell-donation-are-costing-lives, Accessed September 25, 2023. The U.S. bone marrow registry’s website lists the following “myths”: “Donating bone marrow involves opening up or removing bones”; “Some think that the only way to donate blood stem cells is through a surgical procedure”; “Donation is very painful”; “Discomfort and side effects vary from person to person, but it’s nothing like what you see on TV” https://bethematch.org/support-the-cause/donate-blood-stem-cells/myths-and-facts-about-blood-stem-cell-donation/, Accessed September 25, 2023.

3 Similarly, the website of the U.S. bone marrow registry reports that “about 90% of donations are peripheral blood stem cells”; see https://bethematch.org/support-the-cause/donate-blood-stem-cells/myths-and-facts-about-blood-stem-cell-donation/.

4 For the webpage addressing German donors, see https://www.dkms.de/rund-um-die-spende. An equivalent English version addressing people living in the UK is: https://www.dkms.org.uk/donation-explained/stem-cell-donation.

5 Survey participants were asked to allocate 100 points between these four items (with each point representing one percent) and could only proceed to the next survey question if the sum of the four percentage values added up to 100.

6 See, for instance, https://www.dkms.de/faq, section “*Fragen zur Spende: Wie läuft eine Stammzellspende ab?*", or https://www.dkms.org.uk/faq, section “*How are blood stem cells collected?*", both retrieved on March 19, 2024.

7 For the slogan, see https://www.dkms.de/informieren/medizin-forschung/so-wirst-du-stammzellspender-in and https://www.dkms.de/aktiv-werden/kampagnen-aktivitaeten/blutkrebs-besiegen.

8 As we have elicited the stated willingness to donate in case donors are medically eligible and available for donation, this effect size should be interpreted as upper boundary of the true effect size.

9 In Table 4 in the Appendix, a potential donor’s willingness to donate (next week and next year) is regressed on respondents’ beliefs regarding the likelihood of each extraction method separately in each column. Columns 1 and 3 indicate that donors who assign a higher likelihood to stem cell extraction from iliac crest and peripheral extraction, respectively, are significantly more willing to donate stem cells in the next week. On the contrary, assigning higher likelihoods to spine and cheek as extraction methods, which never happen at DKMS, are significantly associated with a reduced willingness to donate stem cells. The effect sizes are economically significant, with magnitudes ranging between 1.2 and 5.4 percentage points. The results are robust to using the intention to donate in one year as the outcome.

10 In a sensitivity check, results are qualitatively similar if we set the threshold to $60\%$ in Table 11 in Appendix A.
